# Understanding Pain and Agitation Through System Analysis Algorithms in People With Dementia. A Novel Explorative Approach by the DIGI.PAIN Study

**DOI:** 10.3389/fpain.2022.847578

**Published:** 2022-03-17

**Authors:** Bettina S. Husebo, Maarja Vislapuu, Malgorzata A. Cyndecka, Manal Mustafa, Monica Patrascu

**Affiliations:** ^1^Department of Global Public Health and Primary Care, Centre for Elderly and Nursing Home Medicine, University of Bergen, Bergen, Norway; ^2^Department of Nursing Home Medicine, Bergen, Norway; ^3^Municipality of Bergen, Faculty of Law, University of Bergen, Bergen, Norway; ^4^Oral Health Centre of Expertise in Western Norway, Bergen, Norway; ^5^Complex Systems Laboratory, Department of Automatic Control and System Engineering, University Politehnica of Bucharest, Bucharest, Romania

**Keywords:** dementia, pain, behavior, agitation, algorithms, systems

## Abstract

**Background:**

Many people with dementia (PwD) live and die with undiagnosed and untreated pain and are no longer able to report their suffering. Several pain assessment tools have been developed, tested, and implemented in clinical practice, but nursing home patients are reported to be still in pain. Clinicians and research groups worldwide are seeking novel approaches to encode the prediction, prevalence, and associations to pain in PwD.

**Participants:**

The data in this analysis are acquired from the COSMOS study, a cluster-randomized controlled trial (2014 to 2015), aimed to improve the quality of life in nursing home patients (*N* = 723) through the implementation of a multicomponent intervention. We utilize baseline data of PwD (*N* = 219) with complete datasets of pain and agitation.

**Method:**

Systems analysis explores the relationship between pain and agitation using the Mobilization-Observation-Behavior-Intensity-Dementia (MOBID-2) Pain Scale, Cohen-Mansfield Agitation Inventory (CMAI), and Neuropsychiatric Inventory-Nursing Home version (NPI-NH). For each patient, the individualized continuous time trajectory, and rates of change of pain and agitation are estimated. We determine the relationship between these rates by analyzing them across the entire group.

**Results:**

We found that the new analysis method can generate individualized estimations for pain and agitation evolution for PwD, as well as their relationship. For 189 of 219 PwD, results show that whenever pain increases or decreases, agitation does too, with the same rate. The method also identifies PwD for whom pain or agitation remains constant while the other varies over time, and patients for whom agitation and pain do not change together. The algorithm is scalable to other variables and compatible with wearable devices and digital sensors.

**Conclusion:**

We presented a new approach to clinical data analysis using systems concepts and algorithms. We found that it is possible to quantify and visualize relationships between variables with a precision only dependent on the precision of measurements. This method should be further validated, but incipient results show great potential, especially for wearable-generated continuous data.

## Introduction

Dementia reduces the total life expectancy of patients (1). However, patients do not necessarily die from the disease, but rather *with* it (2). The life expectancy after diagnosis is on average 4.5 years but can extend to 11 years, depending on the patient's age at the time of diagnosis and the presence of comorbidities, such as hypertension or diabetes (2). For patients with dementia (PwD), the end-of-life period is considered to be their final stage, beginning with the admission to the nursing home after diagnosis; on average, end-of-life expectancy is 2.5 years (3). Studies on pain and palliative end-of-life care, particularly for nursing home patients with dementia, underline that many PwD live and die with behavioral and psychological symptoms (BPSD) (e.g., psychosis, agitation, and depression), and undiagnosed and untreated pain (4). The main reason for this symptom load is that PwD are no longer able to report their suffering, the effect of medication after treatment has been initiated or potential side effects of the treatment. This is especially true for the assessment and management of pain because PwD are unable to describe the intensity, location, and duration of their pain experience (5, 6). Acute and chronic pain in nursing home PwD is often related to musculoskeletal diseases, multimorbidity, infections (e.g., urinary, oral, etc.), or injuries, and ~90% have chronic pain that lasts for three months or longer (7–9). For instance, oral infections might cause unobserved pain by proxy-raters (10, 11). Although recommendations to screen orofacial pain routinely exist, the pain assessment reliant on proxy-rater scales was found to be challenging (12, 13).

Importantly, pain is a critical trigger for underlying BPSD such as agitation and aggression, depression, apathy, and eating and sleeps disturbances (14). The PAIN.BPSD trial demonstrated that individual pain treatment reduces agitation, depression, and sleep disturbances, and may help alleviate eating disturbances (6, 15–18). However, pain management must be conducted with caution. In a placebo-controlled trial on buprenorphine, our group revealed that PwD have a reduced opioid tolerability probably due to anticholinergic side effects (19–21). Despite potential adverse events, 30% of Norwegian and 40% of Danish nursing home patients receive opioids (21).

To improve the compound challenges, several pain tools have been developed, tested, and implemented in clinical practice. The Mobilization-Observation-Behavior-Intensity-Dementia (MOBID-2) Pain Scale, is one such proxy-rater, validated instrument for PwD to improve the treatment of pain (5, 6, 9, 22). Meanwhile, nursing home patients are reported to be still in pain because such tools are barely utilized in PwD, and they are often excluded from clinical trials (23–25). A crucial bottleneck is the staff's ability to recognize that the PwD is in pain at the end of life. The prospective, cross-sectional REDIC study demonstrated that despite increased drug use 46% were in pain, 53% had dyspnea, and 31% anxiety (4). The COSMOS trial, a multicomponent, cluster randomized controlled intervention including nursing home patients with and without dementia, shows a pain prevalence of 46% and 23% had agitation. This in mind, clinicians and research groups worldwide are seeking novel approaches and methods to encode the prediction, prevalence, and associations to pain in PwD. We aim to describe an innovative way to analyze pain and agitation data based on our recent experiences by algorithm developments, integrating the exploratory systems analysis with the statistical one.

Research suggests that data acquired from the mapping of a person's physical activity and rest, including steps walked, hours of sleep, and amount of time spent sitting vs. lying down vs. standing, can serve as a marker for a number of clinical conditions, including for instance pain and agitation (26–29). Inspired by these perspectives and despite the fact that our data are not yet device generated, we aim to present a method on algorithms utilizing pain and agitation data from the COSMOS trial to predict and present associations in a different manner. We hypothesize that:

(1) the individualized estimation of pain evolution over time for PwD is possible using system analysis algorithms.
(2) the individualized estimation of agitation evolution over time for PwD is possible using system analysis algorithms.
(3) the relationship between pain and agitation for PwD can be identified and visualized using system analysis algorithms.
(4) the identified relationship can be modeled into a set of equations useful for monitoring the effect of medication use in PwD.

## Methods

The data in this analysis are acquired from the COSMOS study, a cluster-randomized controlled trial (2014 to 2015), aimed to improve the quality of life in nursing home patients through the implementation of a multicomponent complex intervention. COSMOS is the acronym for the intervention on COmmunication, Systematic assessment and treatment of pain, Medication review, Organization of activities, and Safety. The study included 723 patients from 67 different nursing home units in Western and Eastern of Norway. The study protocol and some results of the COSMOS intervention are published elsewhere (30, 31). Patients were included if they were ≥65 years and have stayed at the nursing home for at least 2 weeks. Patients with a life-expectancy <6 months were excluded from the study. In these analyses, we utilize baseline data of 219 people with a complete dataset on pain and agitation.

### Outcome Measures

The ratings of pain and BPSD were made by a trained research assistant based on a face-to-face interview with the caregiver who is familiar with the patient. Pain was assessed using the MOBID-2 Pain Scale which has thoroughly been tested for validity, reliability, and responsiveness including nursing home patients with dementia (9, 32). The tool consists of two parts, where part 1 assesses musculoskeletal pain through five actively guided movements where raters (nursing home staff) are encouraged to look for pain behavior in the patient during the movements. Part 2 also consists of five items that assess pain coming from head, skin, and internal organs. For each item, raters assess the patient's pain on a NRS from 0 to 10, where 0 represents no pain at all, while 10 represents the worst pain possible. Finally, raters take all assessments into account and suggest the patients' total pain score on a NRS from 0 to 10. A total pain score ≥3 is viewed as clinically significant pain.

The Cohen-Mansfield Agitation Inventory (CMAI) is a 29-item instrument (score range: 29–203) to assist caregivers in rating the frequency of manifested agitation and other behavioral disturbances in nursing home patients with dementia (33). CMAI items are rated on a 1–7 point scale of frequency, ranging from never [1], occurring less than once a week [2], once or twice a week [3], several times a week [4], once or twice a day [5], several times a day [6], or several times an hour [7], respectively. Good validity and reliability of the CMAI have been reported. Factor analyses demonstrate that agitation is a construct consisting of behaviors that tend to co-occur within individuals (34).

The agitation symptom was also assessed by the Neuropsychiatric Inventory—Nursing Home version (NPI-NH), which was previously translated to Norwegian and tested for validity and reliability (35). The NPI-NH measures the frequency and severity of 12 different neuropsychiatric symptoms such as agitation, depression and psychosis in the last week prior to assessment. Frequency (F) is measured on a scale from 0 to 4, where 0 represents symptom not present, and 4 represents symptom present daily. Severity (S) is measured on a scale from 1 to 3, where 1 represent mild symptom severity with little stress on the patient, and 3 represents a severe symptom with high stress on the patient. The score for frequency and severity (F x S) are then multiplied to generate a score for each symptom ranging from 0 to 12. A F x S score ≥4 is considered a clinically significant symptom (36).

### Concept Description for the Data Analysis

Exploratory system analysis is a core component of modeling, with the goal of identifying its behavior, components, or purpose. In this case, the studied system is biological in nature, and presents a multitude of interconnected components with strong interdependencies, making it near impossible to explore analytically. Therefore, we choose a data-driven approach.

Pain and agitation can be measured, and studies reveal that treatment of pain reflects on levels of agitation (14, 17, 37). However, the human body displays non-holonomic behavior as a system (future states are dependent on present and past states), and instant measurements (performed at a specific moment in time) only tell part of the story, as they cannot produce information about the state of the patient, past or future. Hence, we must introduce the time dimension in the analysis and for this, we look at the trajectories of pain and agitation. In this paper, we define the *trajectory* as the evolution of a variable over time.

**Figure 1:**
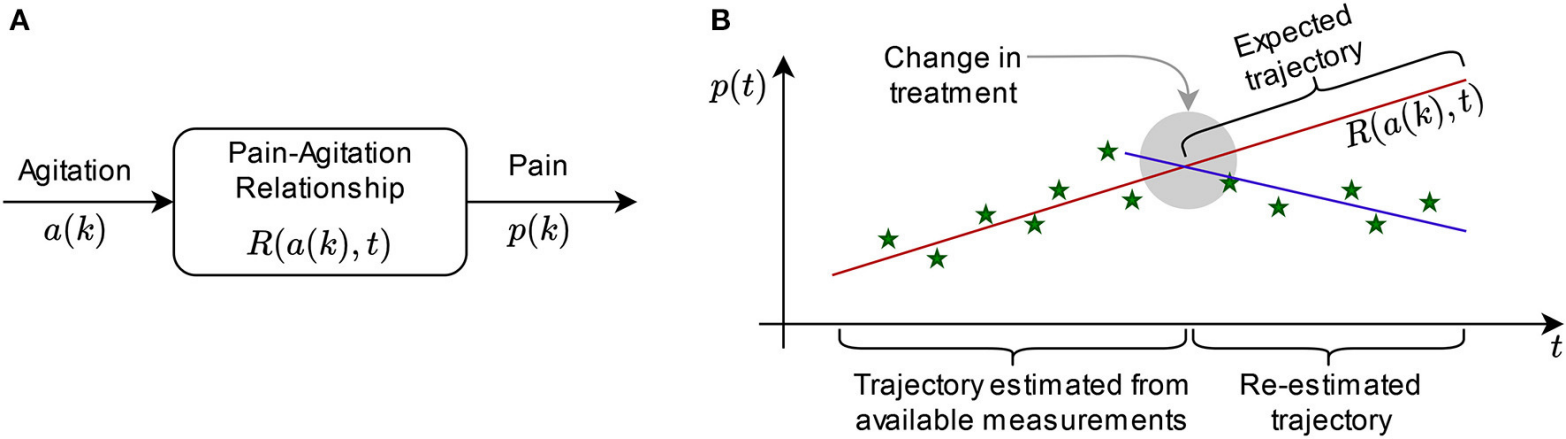
Concept: **(A)** Illustration of input and output variables and the relationship between them for system analysis; **(B)** exemplification of the sliding windows in prediction: the trajectory estimated from measurements generates the expected trajectory (prediction), but it is re-estimated if the new measurements do not match the expectation, while the difference in behavior can be interpreted by a medical expert.

In this paper, we explore how pain and agitation are connected, and in a system context, two options are open: estimation of pain from agitation levels, or vice-versa. We begin by exploring the former. For this, we choose agitation as the input (independent) variable, pain as the output (dependent variable), and focus on the relationship between pain and agitation (Figure 1A). A bidirectional dependency is observable when the relationship is reversible, meaning that for each pain score, we can estimate the agitation score most likely associated with it. The same analysis we present here is viable for the reverse (when pain is the input variable and agitation is the output).

Let *p*(*k*) be the pain score trajectory of a patient over discrete time *k*, and let *a*(*k*) be the agitation score trajectory of a patient over discrete time *k*. Our goal is to find a relationship *R* so that *p*(*k*) = *R*[*a*(*k*)*, t*] over continuous time *t*, which describes the dependency between the trajectories of pain and agitation. While variables *p* and *a* are discrete (instant measurements are performed at certain moments in time), *R* is continuous over time, meaning that it can compute estimations of pain from agitation scores even for the periods between instant measurements.

Relationship *R* can also serve as an estimator for the expected evolution of pain and agitation over time (Figure 1B). For each patient, consider a set of collected data of length *q* [i.e. *q* instant measurements of *p*(*k*): {*p*(1*s*_*t*_), *p*(2*s*_*t*_), …, *p*(*qs*_*t*_)} where *s*_*t*_ is the sampling time, for instance 4 months, and the corresponding set of *q* measurements of *a*(*k*)] have been measured. We determine *R* in continuous time for samples 1 to *q* by adjusting the parameters that describe it. Thus, we can choose any moment in time to produce an estimation of pain vs. agitation. However, this sort of prediction is only accurate for a window of time of maximum *q*/4 in length (38). In practice, the accuracy can only be trusted for a specified interval after the measurement window (39). If this interval is, for instance, equal to one sample, then the prediction is made for *p*[(*q*+1)*s*_*t*_] and *a*[(*q*+1)*s*_*t*_]; for the (*q*+2)-th sample, we re-determine *R* using samples 2 through *q*+1, and so on. This way, expected trajectories can be compared with the actual evolution of the variables, being useful to ascertain effects of treatment, for instance, by the medical expert (Figure 1B).

The challenge is to determine a structure for *R* that can reflect the dependencies between input and output. In cases where analytical approaches are viable, *R* is a set of equations with adjustable parameters. In this paper, however, we must determine the shape of *R* through exploratory analysis.

For a PwD, their condition changes over time as an effect of the neurological disorder, medication, etc. and comparing instant measurements does not reveal information about the trend of these changes. To obtain *R* with capabilities of future state estimation, it is important to analyze how pain and agitation evolve, and we introduce the *rate of change* as the first order derivative of the trajectories. Let *p*(*t*) and *a*(*t*) be the continuous time estimates of discrete trajectories *p*(*k*) and *a*(*k*). Then, ṗ(*t*) = d*p*(*t*)/d*t* is the rate of change for pain (*pain rate*), while ȧ(*t*) = d*a*(*t*)/d*t* is the rate of change for agitation (*agitation rate*), where d is the first order derivation operator.

In the case of pain and agitation in PwD, we obtain *R* by comparing the pain rate and the agitation rate, thus describing *the change in pain relative to the change in agitation* as ṗ(*t*) = *V*[ȧ(*t*)] where V is defined in continuous time. Thus, *p*(*t*) = ∫*V*[ȧ(*t*)]*dt*. Of note is that for numerical computation, initial conditions are unknown and the discrete interval computation is dependent on the number of samples (for each participant). *R* is now the result of the integration on *V* and the problem of finding *R* is reduced to finding *V*.

Determining *V* in this analysis is preferred to determining *R*, because this way we ensure the temporal dimension is included in *R*, and that the representation of *R* remains non-holonomic. This approach allows us to ascertain the way the trajectories of pain and agitation co-evolve over time, enabling the estimation of future states.

To find *V*, we explore the dataset of length *q* of each patient. For each *q* instant measurements of pain scores *p*(*k*) and agitation scores *a*(*k*), we estimate the approximations *p*(*t*) and *a*(*t*) by first normalizing the measurements and then fitting a suitable model over the data. In this paper, we begin this exploration with polynomial models for *p*(*t*) and *a*(*t*), without discarding the inclusion of non-linearities in future work.

Thus, let *p*(*t*) = *p*_0_ + *p*_1_*t* + *p*_2_*t*^2^ + ... + *p*_*m*_*t*^*m*^ and *a*(*t*) = *a*_0_ + *a*_1_*t* + *a*_2_*t*^2^ + ... + *a*_*n*_*t*^*n*^. By adjusting parameters *p*_0_-*p*_*m*_, *a*_0_-*a*_*n*_ and orders *m* and *n*, we fit the polynomials over the measured data. As a rule, *m* and *n* should not be larger than the dataset length *q*. Zero-order polynomials (*m*=*n*=0) would mean that pain and agitation do not vary over time, therefore *m, n* > 0.

For the first-order polynomials (*m*=*n*=1), *p*(*t*) = *p*_0_ + *p*_1_*t* and *a*(*t*) = *a*_0_ + *a*_1_*t*, which give ṗ(*t*) = *p*_1_ and ȧ(*t*) = *a*_1_. With these two variables, we can explore commonalities across the patient group without losing the temporal dimension (it is implicit) and determine *V* as the relation between *p*_1_ and *a*_1_. Using regression analysis, for instance, *V* would take the form of a linear first-order polynomial approximation:


(1) $$\begin{array}{r} ṗ\left(t\right)=V\left[ȧ\left(t\right)\right]=v_{0}+v_{1}ȧ\left(t\right)+v_{2}{ȧ}^{2}\left(t\right)+...+v_{\text{z}}{ȧ}^{z}\left(t\right)\Rightarrow \\ \;p_{1}=v_{0}+v_{1}a_{1}+v_{2}a_{1}^{2}+...+v_{\text{z}}a_{1}^{\text{z}} \end{array}$$

## Ethics

The present study uses discreet observation measures that allow for collecting data without a direct elicitation of information from PwD. From the ethical point of view, the deployment of such measures is crucial as it excludes the necessity of triggering pain by pain stimuli, which is to be assessed. Moreover, the present study relies on data that have already been collected for the COSMOS trial while the assessment of pain falls within the aims sought by the COSMOS study.

In some cases, the use of observation measures may raise privacy and ethical concerns as such measures often involve collecting data and observations without the knowledge of the research participants. This is, however, not the case in the present study. Informed consent was obtained in written and verbal form from patients with the cognitive ability to understand the information regarding the COSMOS-study. For patients lacking this ability, presumed consent was obtained, after explaining the study procedure, from the patients next of kin or legal guardian. The trial was approved by the Regional Committee for Medical and Health Research Ethics, West Norway (REK 2013/1765) and registered at clinicaltrials.gov (NCT02238652).

## Results

We demonstrate the analysis concept by applying it to anonymized data from the COSMOS study (30, 31). All algorithms have been written and executed using the Matlab^®^ framework (40). The mean age of the selected group of patients is 86.2 years (± 7.2) and 75.8% are female. The median Mini-Mental State Examination (MMSE) score is 11 points. We select all patients (*N* = 219) with complete measurement sets over the 9 months of the COSMOS study, and obtain patient measurements available for pain and agitation scores, in three datasets:

- Total pain scores *p*(*k*) evaluated with the MOBID-2 scale, with minima and maxima at [*p*_*min*_, *p*_*max*_] = [0, 10].
- Total agitation scores *c*(*k*) evaluated with the CMAI scale, with theoretical extrema at [29, 203], but for the present study with measured minima and maxima for the patient group at [*c*_*min*_, *c*_*max*_] = [29, 123].
- Sub-item agitation scores for frequency and severity *a*(*k*) evaluated with the NPI-NH scale, with minima and maxima at [*a*_*min*_, *a*_*max*_] = [0, 12].

Each patient dataset has *q* = 3 measurements, with *k* ϵ {0, 4, 9} [*month*]. Out of *N* = 219, for *N*_*P*_ = 196 patients the MOBID-2 pain score was >0 at least once, and for *N*_*T*_ = 107 patients the MOBID-2 pain score was >0 on all measurements.

### Normalization

Data is normalized within the [0, 100] [*pcnt*] interval (corresponding to percentages as measuring unit, notation *pcnt*): $normalizedValue\;\left[pcnt\right]=\frac{\left(measuredValue\;\left[scale\;unit\right]-minima\right)\cdot 100}{\left(maxima-minima\right)}$, resulting the normalized datasets of *p*_*z*_(*k*), *a*_*z*_(*k*), and *c*_*z*_(*k*).

*Continuous time estimation* is performed using least-squares polynomial fitting. Due to length *q* = 3, the maximum feasible order of the polynomial is 1, resulting in first-order approximations over 9 months, with sampling times at 0, 4, and 9 months respectively:

- {*p*_*z*_(month 0), *p*_*z*_(month 4), *p*_*z*_(month 9)} → *p*(*t*) = *p*_0_ + *p*_1_*t*
- {*c*_*z*_(month 0), *c*_*z*_(month 4), *c*_*z*_(month 9)} → *c*(*t*) = *c*_0_ + *c*_1_*t*
- {*a*_*z*_(month 0), *a*_*z*_(month 4), *a*_*z*_(month 9)} → *a*(*t*) = *a*_0_ + *a*_1_*t*

All polynomial parameters are individual to each patient, resulting in 219 × 3 = 657 polynomials, with *p*_0_ = *p*_*z*_(month 0), *c*_0_ = *c*_*z*_(month 0) and *a*_0_ = *a*_*z*_(month 0) as the baselines for each patient.

### Rates of Change

Derivation of polynomials *p*(*t*), *a*(*t*), and *c*(*t*) yields the rates of change as *p*_1_, *a*_1_, and *c*_1_, for each of the 219 patients in the group. Rates of change are measured in *pcnt/month* (percentage per month). A rate of change approximately zero, means the pain or agitation scores are constant; positive rates of change indicate increase in pain or agitation, while negative rates of change indicate decrease. For exemplification, a rate of change of 20 [*pcnt/month*] in pain means that the pain score of the patient increases by 2 points on the MOBID-2 scale every month, while a rate of change of −10 [*pcnt/month*] means that the pain scores decrease by 1 point every month. Considering the extrema of each scale and the significance of the *pcnt/month* measuring unit for rates of change, we round the rates of change results to nearest integers toward zero.

### Determining the Relationship Between Pain Rates and Agitation Rates

We begin by computing the correlation between pain and agitation rates across the patient group to ascertain if the relationship exists in this patient group for these datasets. Results show significant correlations between:

- *p*_1_ and *c*_1_ (219 pairs), with a *p*-value of 0.0264 and a correlation coefficient of 0.15.
- *p*_1_ and *a*_1_ (219 pairs), with a *p*-value of 0.069 and a correlation coefficient of 0.1231.

**Figure 2:**
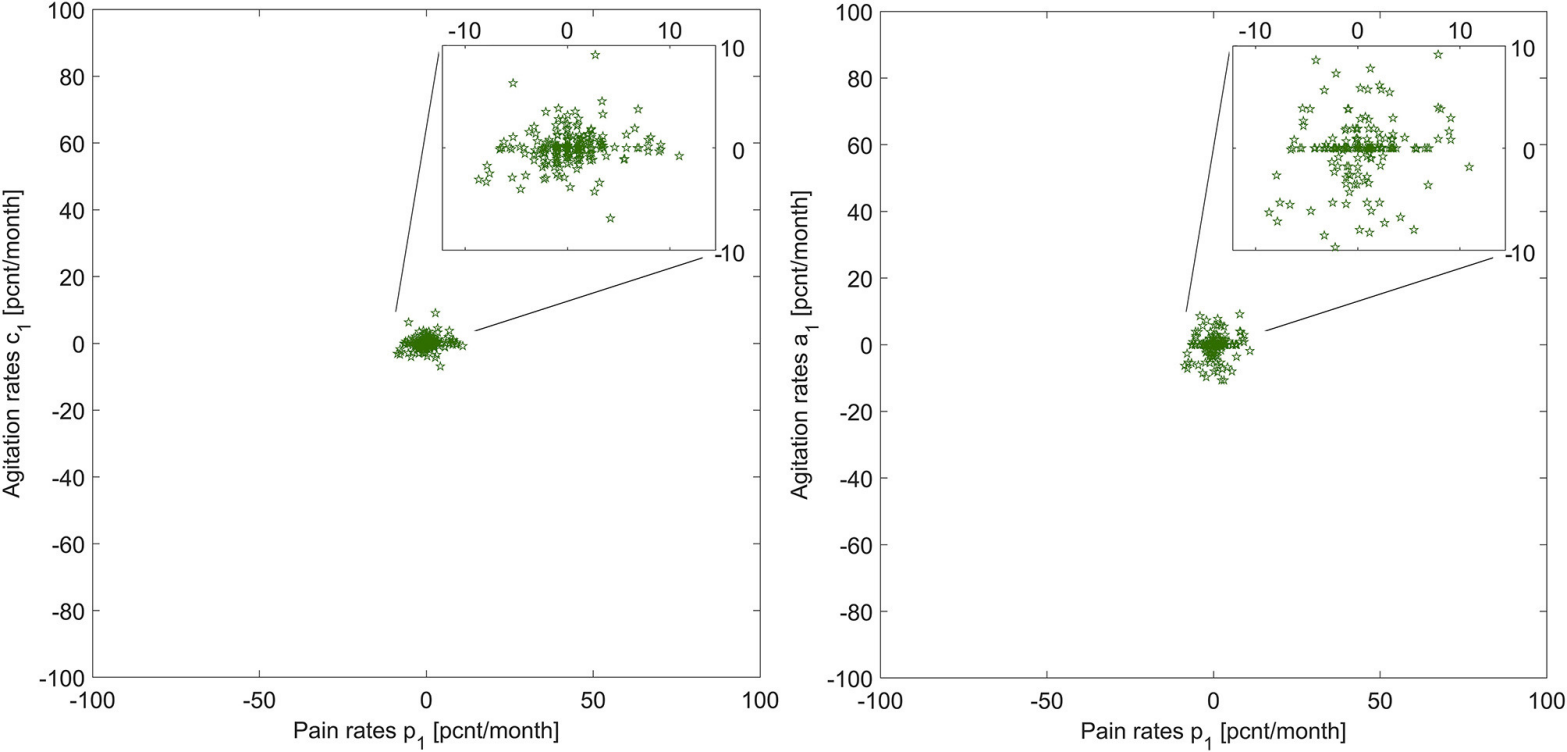
Illustration of pain rates vs. agitation rates for the two agitation indicators, for all patients.

Figure 2 shows the graphic of *p*_1_ vs. *a*_1_, and *p*_1_ vs. *c*_1_ across the patient group (*N* = 219). This visualization helps us understand what relationship *V* might look like for this group: pain and agitation evolve together, at similar rates. The next step in this explorative analysis is to investigate if we can model and describe this relationship.

### Analyzing the Relationships and Their Outliers

To quantify the similarities between rates of change in pain and agitation scores, we observe that patients can have:

- matching rate of change, within a 5 [*pcnt/month*] margin (we choose this margin based on significance relative to measurement extrema and to account for measurement errors), which can be: constant (within a margin of 1 [*pcnt/month*]), increasing, or decreasing;
- different rates of change with more than 5 [*pcnt/month*] between pain and agitation, which can be increasing (both or one increasing one constant) or decreasing (both or one decreasing one constant);
- opposite rates of change with a difference of more than 5 [*pcnt/month*] between pain and agitation, but where one is increasing, and one is decreasing.

**Figure 3:**
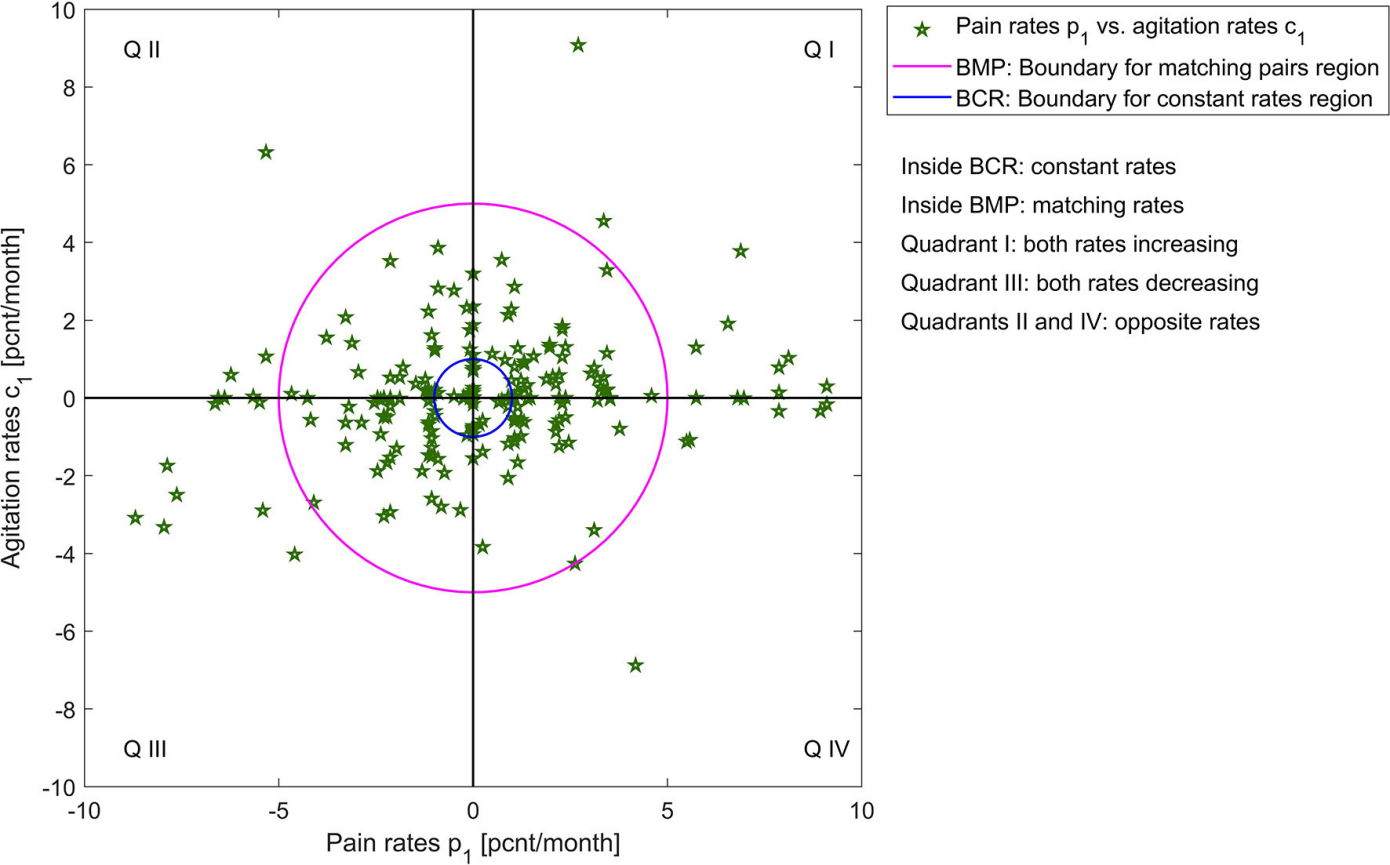
Visualization of pain rates *p*_1_ vs. agitation rates *c*_1_.

**Figure 4:**
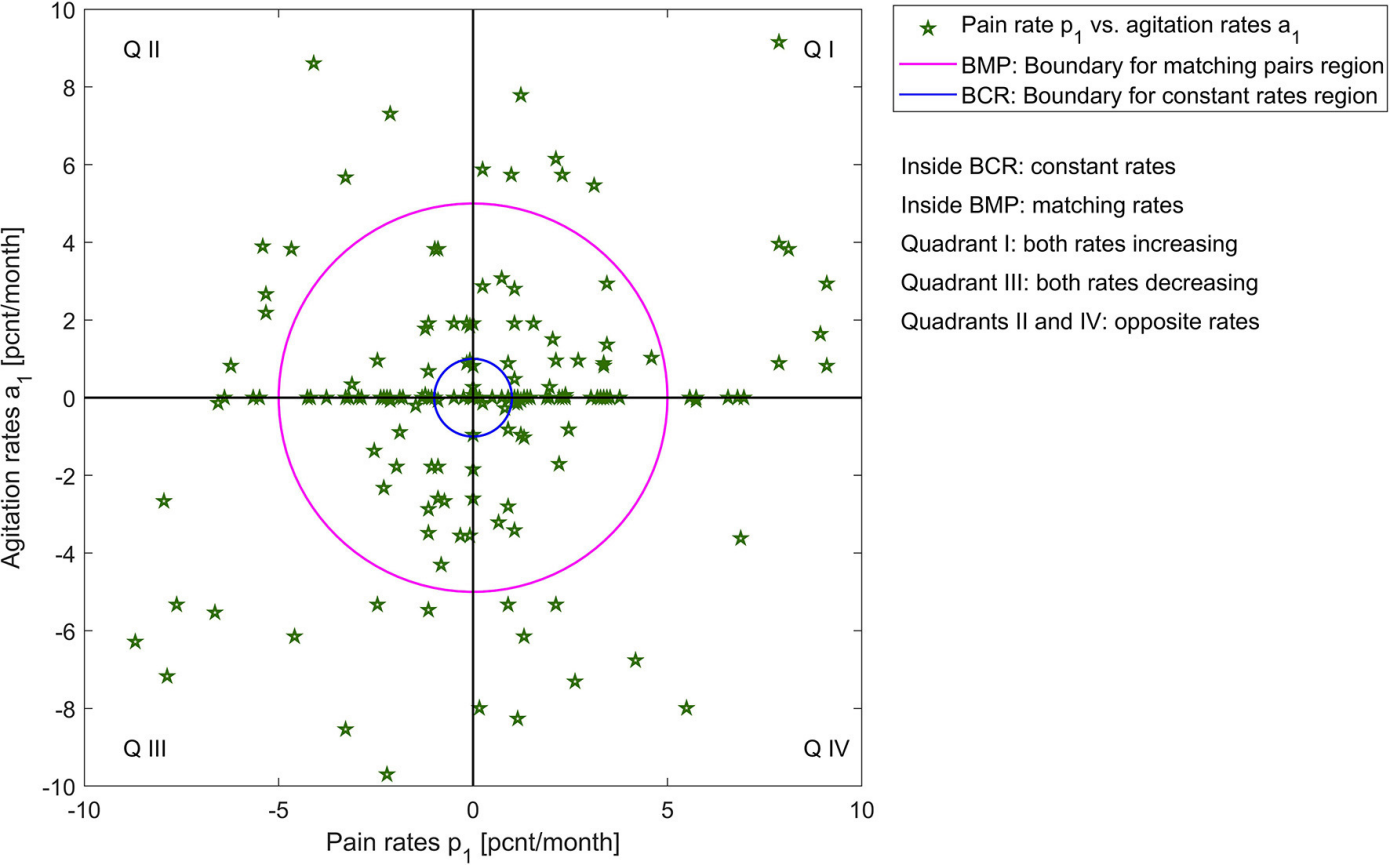
Visualization of pain rates *p*_1_ vs. agitation rates *a*_1_.

A visualization of these categories for both agitation indicators vs. pain rates is presented in Figures 3, 4; Supplementary Materials 1, 2.

For each patient, the relationship between pain and agitation is given by Equation 1. Knowing *p*_1_, *c*_1_ and *a*_1_, we must determine the coefficients of *V*(*t*), notating *V*_*c*_(*t*) for total agitation scores, and *V*_*a*_(*t*) for the sub-item.

Table 1 shows the percentages of these categories out of the entire patient group. Many patients have similar (matching, within a 5 [*pcnt/month*] margin) rates of change. For these patients with matching rates, we obtain significant correlations:

- *p*_1_ vs. *c*_1_ (189 pairs), with a *p*-value of 0.000446 × 10^−5^ and a correlation coefficient of 0.3861.
- *p*_1_ vs. *a*_1_ (187 pairs), with a *p*-value of 0.000192 × 10^−11^ and a correlation coefficient of 0.5303.

**Table 1:** Category percentages.

	**Matching rates**			**Different rates**		**Opposite rates**
	**Constant**	**Increasing**	**Decreasing**	**Increasing**	**Decreasing**	
Pain rates *p_1_* vs. Agitation rates *c_1_*						
Patients out of total	189 (86.3%)			16 (7.31%)		14 (6.39%)
	106 (48.4%)	45 (20.55%)	38 (17.35%)	11 (5.02%)	5 (2.28%)	
Pain rates *p_1_* vs. Agitation rates *a_1_*						
Patients out of total	187 (85.39%)			13 (5.94%)		19 (8.67%)
	98 (44.75%)	44 (20.09%)	45 (20.55%)	8 (3.65%)	5 (2.28%)	

This result shows that for 189 of the 219 patients, whenever pain increases or decreases, agitation does too, *with the same rate*. We conclude that for this subgroup, *p*_1_ = *c*_1_ and *p*_1_ = *a*_1_, which gives us: *V*_*c*_[ċ(*t*)] = *c*_1_ and *V*_*a*_[ȧ(*t*)] = *a*_1_. Thus, the relationships between pain and the two rates of the agitation scores are:


(2) $$\begin{array}{r} p\left(t\right)=\int V_{c}\left[ċ\left(t\right)\right]dt=c_{1}t+C\text{ and}\\ p\left(t\right)=\int V_{a}\left[ȧ\left(t\right)\right]dt=a_{1}t+A,\forall C,A\in \mathbb{R}, \end{array}$$

where *C* and *A* are the constants of integration, dependent on initial conditions. In the setting of this study, *C* and *A* can be determined from baseline measurements. If *p*_0_ is known, then *C* = *A* = *p*_0_. However, Equation 2 illustrates that the change in pain can be estimated from the change in agitation for any initial condition of pain, even the null condition, i.e., the assumption that the patient had no pain at baseline. The advantage is that even when choosing a different moment to begin the analysis or measurement, this new baseline of pain score does not affect parameters *c*_1_ and *a*_1_, which are obtained from agitation. Finally, from *c*(*t*) = *c*_0_ + *c*_1_*t* and *a*(*t*) = *a*_0_ + *a*_1_*t* we can now write:


(3) $$\begin{array}{r} p\left(t\right)=c\left(t\right)+\left(C-c_{0}\right)\text{ and }p\left(t\right)=a\left(t\right)+\left(A-a_{0}\right),\forall C,A\in \mathbb{R} \end{array}$$

Changes in agitation are reflected by the changes in pain relative to the baseline.

Results also show those patients for which pain or agitation remains constant while the other varies over time, as well as those patients for which the agitation and pain do not change together (i.e., when one increases and the other decreases). For these, each *V*_*c*_(*t*) and *V*_*a*_(*t*) must be computed individually (obtaining *p*_1_ as a function of either *c*_1_ or *a*_1_), because conclusions across these subgroups are not generalizable. Similarly, these equations will not be dependent on baseline measurements. For each patient, consider δ_*c*_ and δ_*a*_ such that *p*_1_ = δ_*c*_*c*_1_ and *p*_1_= δ_*a*_*a*_1_. It follows that:


(4) $$\begin{array}{r} p\left(t\right)=\delta_{c}c\left(t\right)+\left(C-\delta_{c}c_{0}\right)\text{ and}\\ p\left(t\right)=\delta_{a}a\left(t\right)+\left(A-{\delta_{a}a}_{0}\right),\forall C,A\in \mathbb{R} \end{array}$$

Of note, here, is that Equations 3 and 4 are valid for normalized pain and agitation scores, so numerical compatibility exists between the left-hand and right-hand sides of the equations.

## Discussion

The aim of this study was to analyze the utilization of pain and agitation though novel approaches to present their associations in a different manner. As hypothesized, we found that the analysis method and subsequent algorithm we presented in this paper is able to generate individualized estimations for pain and agitation evolution for PwD. The algorithm does not require special calibration for each person during usage and it is compatible with device-generated data. Further, we demonstrated that the relationship between pain and agitation for PwD can be identified and visualized using system analysis algorithms. The method is scalable to other measurements and other groups, such as sleep patterns vs. daily activity cycles. As outcomes, the method produces equations (mathematical models) that can be utilized to monitor the effect of medication use in PwD, by computing expected trajectories of patients (without change in medication) to be compared with measurements taken after change in medication. With enough data, a clinician can assess overall trends of a person's pathway, that are resilient to small fluctuations due to daily contexts. Moreover, large deviations in measurements are useful for the analysis as they point to contexts that require further investigation. For a clinician, this information is important as it reflects on the trajectories of pain and agitation over time.

At this timepoint we only have 219 measures of three data points, but consider, for instance, if we measure agitation daily using a wearable device and the readings for a week give {23, 23, 24, 28, 74, 26, 23} [*pcnt*], we see agitation levels that jump suddenly and then decrease just as suddenly. However, the overall rate of change can still be computed even in the presence of this outlier value (in this example the trend is slightly increasing at 2 [*pcnt*/*day*] vs. an expected value of 1.6 [*pcnt*/*day*] computed from the 4 days prior to the spike), which then can be assessed for meaning (What happened to generate that particular spike? Did medication have an unexpected effect? Or was a different stressor applied, for instance a visitor upsetting the patient? etc.). If the sudden increase persists, it will be reflected in the next window of measurements.

Further, consider for instance a case in which, over two months, agitation and pain rates increase together at 0.5 [*pcnt/month*], showing a relationship in which pain and agitation co-evolve with matching rates, the attending clinician decides to prescribe a regular dosage of analgesic medication. Upon further measurements, the rates of change for agitation are still increasing, estimated at 0.4 [*pcnt/month*], while the pain rates become decreasing, at −0.1 [*pcnt/month*]. This is an indication that (a) the pain medication has the expected effect and (b) that the increasing agitation might have other important causes not related to pain. This information suggests the need for further investigation into the patient's overall status.

In the context of this paper, while agitation alone is not an indicator that pain exists, the analysis we performed shows that it is possible to estimate how much the pain levels have changed from one measurement to the next by measuring agitation, and vice-versa. Further application to different datasets is needed for validation, but the correlation results are promising in supporting this discovery.

Although the number of patients exhibiting the non-matching pain-agitation rates is small relative to the total in the group, the discovered relationships can serve as an indicator for the attending medical staff to check their context: overall state, medication, changes at the end of life, other measurements, etc.

By setting the measurement scales into the group context via their measured extrema vs. theoretical extrema, we observed differences in results. It raises the question whether these differences are significant in a larger context and if scale resolutions are too small or too large, i.e., is there a difference between the CMAI and NPI-NH scales as origins of measurements? The evaluation of the scales does not fall under the scope of this paper, but the results show tremendous opportunity to discover new ways of assessing various measurements tools relative to each other, when their relationships with another measurement is considered. In this case, the CMAI and NPI-NH scales through the relationship with MOBID-2. For a more comprehensive analysis of how CMAI and NPI-NH perform within these algorithms, more measured data points per person are needed.

The numerical results presented in the previous section are subjected to a set of uncertainties, which is expected in exploratory system analysis. From this first analysis, we can derive mitigation measures and requirements for further experiments that would yield higher numerical confidence.

*Normalization of data* is a process meant to remove numerical incompatibilities during analysis. Normalization transforms every numerical measurement into the same measuring unit so that comparisons between values make sense. Comparing, for instance, a score of 4 on the NPI-NH with a score of 4 on the CMAI scale is not possible, because these two scales have different orders of magnitude. Thus, we use the extrema of the scales to transform both into a numerically compatible scale (in this paper we choose the so-called “percentages” that map any measurement scales to a 0–100 interval). When scale native extrema are used in computation, the normalization is viable for any measurement. However, using the entire scale interval might not be relevant for different patient groups, and not all items are actively used, for instance from the CMAI. On the one hand, the native extrema allow for scalability, but have the disadvantage of losing accuracy because the mapping includes measurement intervals for which there is no data. On the other hand, hyper-individualized (as in, person-specific) normalization might be attractive, but it has the disadvantage of high uncertainty: identifying specific extrema for each person is not guaranteed to be valid for any period of time due to changes in the body (caused by any number of factors, from nutritional intake to medication). For this study, we choose the middle-ground, in which we determine group-specific extrema by examining the minima and maxima of all measurements within the group. Looking at results using the theoretical extrema for the CMAI scale [29, 203], we find that *p*_1_ vs. *c*_1_ analysis yields 195 matching pairs with a *p*-value of 0.000608 and a correlation coefficient of 0.2458, which are still significant, but marginally worse than the results for the group extrema, even though the number of pairs is larger. Using the group extrema, results are more consistent with the NPI-NH *for this patient group*. Further investigation is necessary to generate normalization requirements and specification in system analysis.

*Measurement errors* cannot be eliminated, nor quantified. They relate to rater subjectivity and/or bias, be it the caregiver rating or the self-report. The measurement errors affect both the estimations during analysis and the normalization extrema. Minimization of errors is possible by using redundancy of measurement, either by having multiple raters perform the measurement, or using digital tools, such as sensors. The former can put strain on caregiving resources, so we turn to the latter. In our current ongoing work, we are studying the use of wearable devices and the analysis concept presented in this paper will be further tested with new data.

*The dataset length for each individualized estimation* of pain and agitation rates in this paper contains only three measurements taken over 9 months. This decreases the confidence levels in the initial estimations for the rates of change. The procedure, however, is scalable to any dataset length (only limited by the memory capacity and computing power of the processor on which it runs). For further work, we recommend and plan on using much larger individual measurement datasets, with sampling times as low as 1 min, collected over intervals of 1 to 14 days. However, proxy-rater instruments are not suitable for this frequency of data collection, therefore we are evaluating the use of wearable devices and digital sensors to facilitate data collection for these estimations. This may be relevant for PwD at different stages of the disease and also at the end of life.

The prediction accuracy of pain vs. agitation estimation cannot be tested with the data we have at our disposal at the moment because the COSMOS study has ended and the three datapoints of the individualized pain and agitation measurements cannot be divided into two sets, one for generating the prediction and one for validating it.

One of the main objectives of future work is to apply the proposed method to wearable and sensor generated data. We plan on using devices such as Empatica E4, Oura ring, and Fitbit sense to further validate the algorithms. This work is currently in progress.

In what concerns the CMAI scale, we plan on performing analyses using each of 4 factor groups (aggressive behavior, physical non-aggressive behavior, verbally agitated behavior, and hiding and hoarding) to determine their relationship with pain and ascertain whether or not they relate differently to it and to other agitation scales.

In the life of a PwD, pain and agitation, while intricately connected, also relate to other variables, such as cognitive function. During the COSMOS study, data on these variables was collected. The patient's cognitive function was assessed by MMSE, Activities of daily living (ADL) were assessed using the Lawton and Brody ADL assessment tool, while information on medication and diagnoses were obtained from the patient's medical record (41, 42). All assessments were performed by nursing home staff who knew the patient well and received education to use the assessment tools in advance to study start. Our next step is to supplement the analysis by obtaining the relationships between pain, agitation, cognitive function, daily activities, and medication.

## Conclusion

We presented a new approach to clinical data analysis using systems concepts and algorithms. We found that it is possible to quantify relationships between variables with a precision only dependent on the precision of measurements. This method should be further validated, but incipient results show great potential, especially for wearable-generated continuous data.

## Data Availability Statement

The data that supports the findings in this study is subject to restrictions and are available from the corresponding author upon reasonable request.

## Ethics Statement

The trial was approved by the Regional Committee for Medical and Health Research Ethics, West Norway (REK 2013/1765). The patients/participants provided their written informed consent to participate in this study.

## Author Contributions

BH is the primary investigator of the COSMOS from which the data originates. MP designed the study, analyzed the data, and wrote the manuscript together with BH. MV helped with the analysis of the data and reviewed the manuscript. MC contributed with the ethical perspectives regarding PwD, data acquisition, and algorithms. MM added knowledge regarding oral pain in PwD. All authors have read and approved the manuscript prior to publication.

## Funding

This study was funded by The Research Council of Norway (RCN, Sponsor's Protocol Code: 222113) and Rebekka Ege Hegermann's Foundation.

## Conflict of Interest

The authors declare that the research was conducted in the absence of any commercial or financial relationships that could be construed as a potential conflict of interest.

## Publisher's Note

All claims expressed in this article are solely those of the authors and do not necessarily represent those of their affiliated organizations, or those of the publisher, the editors and the reviewers. Any product that may be evaluated in this article, or claim that may be made by its manufacturer, is not guaranteed or endorsed by the publisher.
