# Supplementary material for: Understanding Pain and Agitation Through System Analysis Algorithms in People With Dementia. A Novel Explorative Approach by the DIGI.PAIN Study
Source: Front Pain Res (Lausanne). 2022 Mar 17;3:847578. doi: 10.3389/fpain.2022.847578 (PMC8970316; doi:10.3389/fpain.2022.847578)
Supplement: Supplementary file 1 [file Data_Sheet_1.PDF]

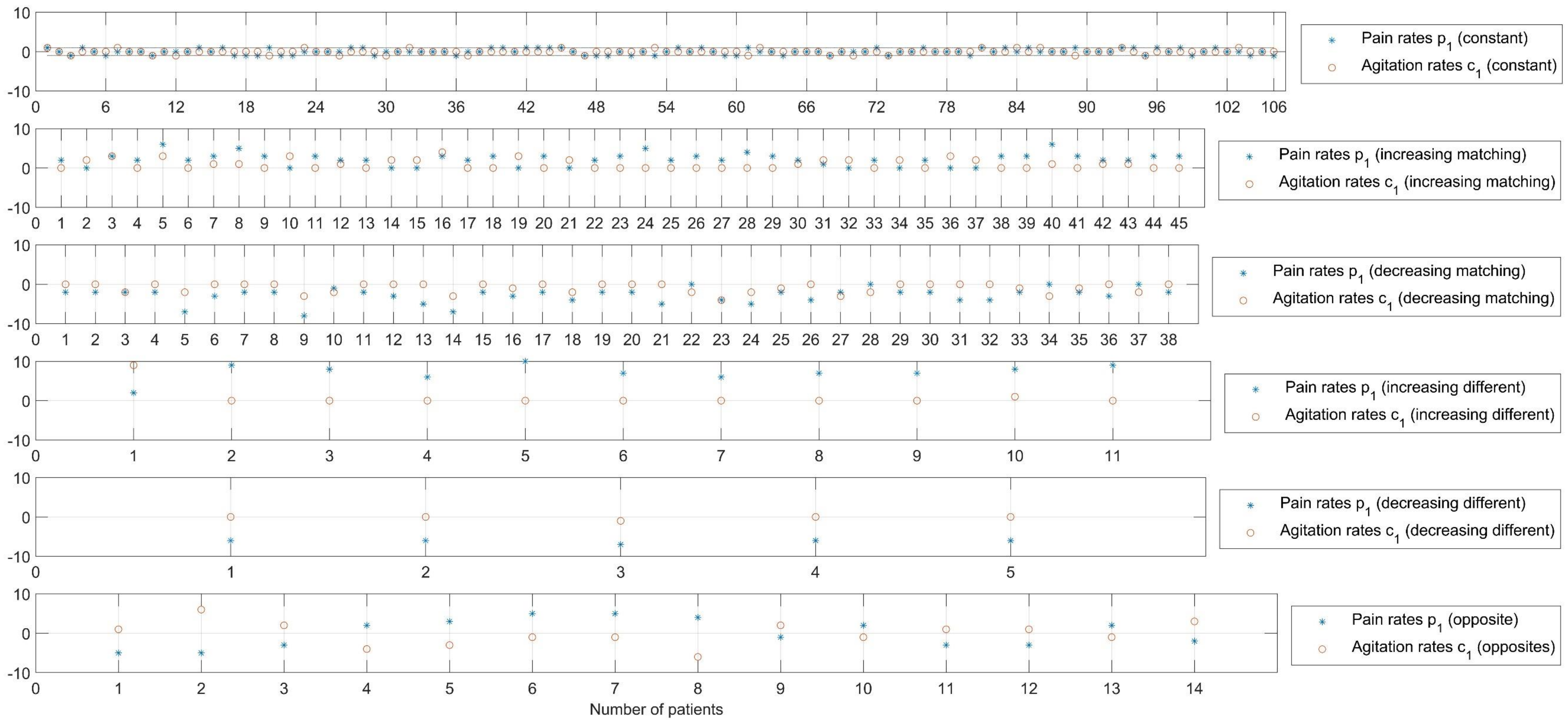

Supplementary 1. Visualization of pain rates p_1 vs. agitation rates c_1 per patient. Both rates are represented on the vertical axis and expressed in [pcnt/month]. The number of patients is represented on the horizontal axis. For clarity, the visualization shows the [-10, 10] [pcnt/month] intervals out of the maximum [-100, 100] [pcnt/month] normalized scales.
